# Supplementary material for: Measuring for primary prevention: An online survey of local community perspectives on family and domestic violence in regional Australia
Source: PLoS One. 2023 Apr 10;18(4):e0284302. doi: 10.1371/journal.pone.0284302 (PMC10085013; doi:10.1371/journal.pone.0284302)
Supplement: Attachment — Submitted filename: PONE paper Reviewer Comments - Vijayarani.docx [file pone.0284302.s001.docx]

***J.Vijayarani,***

*Teaching Fellow,*

*Department of CSE,*

*CEG Campus, Anna University,*

*Chennai 600 025****.***

*Mob:9442816964*

*Email id:* [viji.cs66@gmail.com](mailto:viji.cs66@gmail.com%20)

**Paper Title: " Measuring for primary prevention: an online survey of local community perspectives on family and domestic violence in regional Australia"**

**Paper ID: PONE-D-22-35475**

**Comments:**

This paper proposes an effective way of analyzing the family and domestic violence (FDV) in regional Australia. It is socially relevant to the current scenario. The authors developed questionnaire-based survey to investigate FDV. The questionnaire comprised items on the sociodemographic characteristics of respondents in addition to FDV-related items. The paper is well written. Statistical analysis was performed in a well manner based on the reports from Local Community Attitudes and Exposure to Violence Survey (LCAEVS) and compared with the results of National Community Attitudes to Violence Against Women Survey (NCAS). Exploratory Factor Analysis was performed based on the domain (Knowledge of FDV types and causes, attitudes, personal experiences). This paper needs a minor revision before publication.

1. The analysis of survey data was performed in an effective manner and tabulated. (Table 1- Characteristics of survey respondents, Table 2- Exploratory factor analysis, Table 3- Multiple linear regression, Table 4- Multivariable robust Poisson regression, Table 5 - Dominance Analysis).

2. Data were weighted in respect to the respondents' self-reported gender, highest education level, Indigenous identifier and age group using the ipfweight command in Stata. Any temporal factor has been considered for weight assignment?.

3. Graphs such as Pie charts, Scatter plots and histograms are the most visually appealing survey data analysis methods. Only one type of plot (Fig 1a to Fig 1c) is utilized for visualization. If possible additional plots can be added.

**Other minor issues are,**

1. Check quotes

Uniform quotes pattern should be used. Straight quotes and curly quotes are used in mixed manner.

2. Check references

Uniform formatting should be followed for references. DOIs are missing for more references. Some references are having URLs. References can also be listed in alphabetical order.
